# Supplementary material for: Genome-Wide Analysis of AP2/ERF Gene Superfamily in Ramie (Boehmeria nivea L.) Revealed Their Synergistic Roles in Regulating Abiotic Stress Resistance and Ramet Development
Source: Int J Mol Sci. 2022 Dec 1;23(23):15117. doi: 10.3390/ijms232315117 (PMC9736067; doi:10.3390/ijms232315117)
Supplement: Supplementary file 1 [file ijms-23-15117-s001.zip › Supplementary Figure S1.pdf]

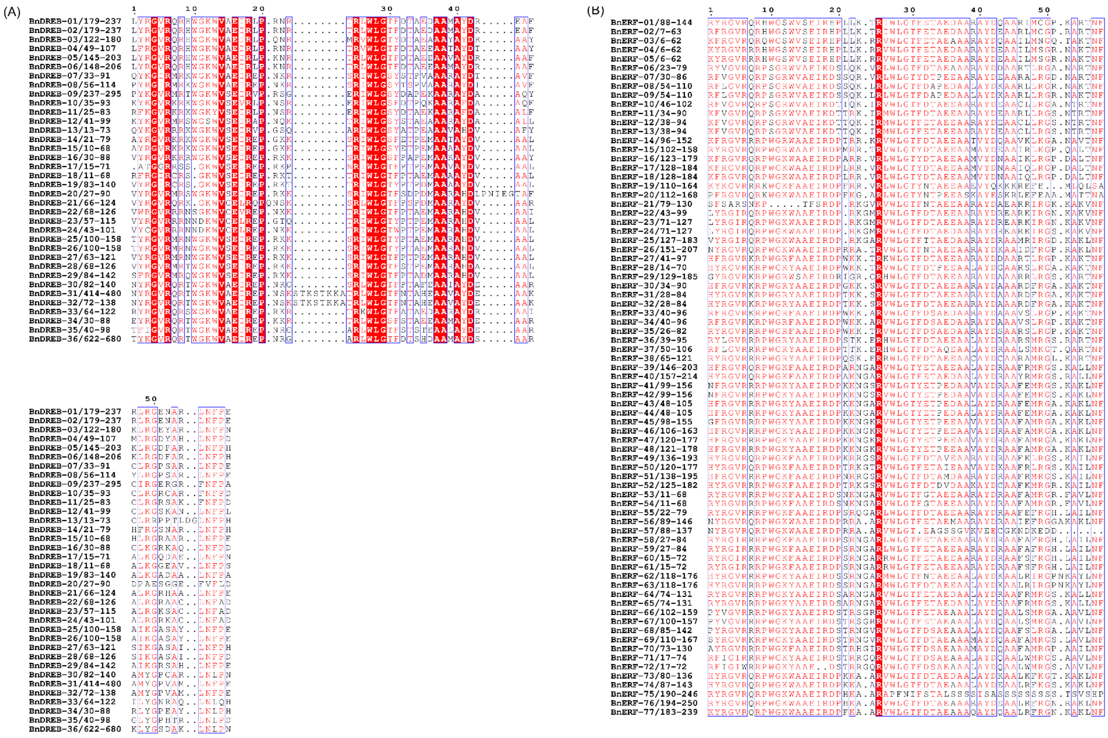

Figure S1. Multiple sequence alignment analysis of conserved domains of ERF subfamily. (**A**) Sequence alignment analysis of conserved domains of BnDREB subfamily. (**B**) Sequence alignment analysis of conserved domains of BnERF subfamily.
